# OP57 Introducing The New MedTech Innovation Scanning Techniques Framework: A Tool For Guiding Horizon Scanning Of MedTech Innovations

**DOI:** 10.1017/S0266462325101153

**Published:** 2025-12-29

**Authors:** Sonia Garcia Gonzalez-Moral, Andrew Mkwashi

## Abstract

**Introduction:**

Horizon scanning (HS) identifies future trends and weak signals, aiding timely healthcare decisions, but lacks a formal framework. The Innovation Observatory’s MedTech Horizon Scanning Techniques (MIST) framework integrates evidence synthesis, HS methods, and foresight approaches. MIST aligns scanning techniques with foresight stages and the technology development pipeline, offering a structured approach to detect emerging science and technology trends for healthcare advancements.

**Methods:**

The MIST framework was developed iteratively, combining HS expertise with regulatory knowledge of medical devices. First, a MedTech development pathway was established with international HS agencies, providing a foundation. Second, the pathway was mapped against the regulatory milestones involved in medical technology development from ideation to post-marketing according to recent European legislation. Last, we overlaid the foresight “Three Horizons Model” with the development stages to produce a framework that combines different innovation stages with their regulatory requirements and maps sources of weak signal detection to the horizons where those sources are most likely to be relevant.

**Results:**

Our MIST framework presents three horizons: emerging, transitional, and imminent. Each horizon maps to different technology readiness levels, life cycle stages, time periods, and regulatory milestones. Each horizon includes guidance on the best-fit HS, information retrieval, and analysis methods. Furthermore, each horizon recommends relevant sources of weak signal detection maximizing the sensitivity of the searches, avoiding unnecessary noise, and achieving efficiencies. The use of the MIST framework is recommended in combination with transparent reporting of the methods and sources used. We have also developed a checklist of reporting items that is available for download in the Open Science Framework (https://osf.io/m39vw/).

**Conclusions:**

The MIST framework represents a stride forward in the direction of HS methods standardization and fulfills a known methodological gap. Our framework aims to underpin robust and systematic HS methods that will add value to the outputs supporting unbiased decision-making. Next steps include its publication and dissemination to facilitate adoption and use by other HS agencies.

